# Supplementary material for: Structural Analysis of Cardanol and Its Biological Activities on Human Keratinocyte Cells
Source: Metabolites. 2025 Jan 30;15(2):83. doi: 10.3390/metabo15020083 (PMC11857407; doi:10.3390/metabo15020083)
Supplement: Supplementary file 1 [file metabolites-15-00083-s001.zip › metabolites-3431062-supplementary.pdf]

Supplementary Table S1: NMR data of the components A, B, and C in cardanol

Atom Position	δ ^{1}H in ppm	^{1}H multipli-city	^{1}H integral	J_{HH} in Hz	δ ^{13}C in ppm	^{13}C multipli-city
**Cardanol with aliphatic chain A (monoene; 42%)**						
1	-				155.33	C
2	6.70	s (b)	1.0		115.40	CH
3	-				145.00; 145.02 [1]	C
4	6.81	d	1.0	7.5	121.04	CH
5	7.18	t	1.0	7.7	129.47	CH
6	6.69	dd	1.0		112.58	CH
1'	2.59	t	2.0	7.7	35.89	CH2
2'	1.63	m	2.2		31.39; 31.40 [2]	CH2
3'	1.36	m			29.08-29.83 [3]	CH2
4'	1.36	m			29.08-29.83 [3]	CH2
5'	1.36	m			29.08-29.83 [3]	CH2
6'	1.36	m			29.08-29.83 [3]	CH2
7'a	2.08	m			27.29; 27.27 [4]	CH2
8'a	5.41	m			129.93; 130.05 [5]	CH
9'a	5.41	m			129.93; 130.05 [5]	CH
10'a	2.08	m			27.29; 27.27 [4]	CH2
11'a	1.36	m			29.08-29.83 [3]	CH2
12'a	1.36	m			29.08-29.83 [3]	CH2
13'a	1.36	m			31.87	CH2
14'a	1.36	m			22.76	CH2
15'a	0.94	t	1.3	7.1	14.23	CH3
**Cardanol with aliphatic chain B (diene; 22%)**						
1	-				155.33	C
2	6.70	s (b)	1.00		115.40	CH
3	-				145.00; 145.02 [1]	C
4	6.81	d	1.00	7.5	121.04	CH
5	7.18	t	1.00	7.7	129.47	CH
6	6.69	dd	1.00		112.58	CH
1'	2.59	t	2.00	7.7	35.89	CH2
2'	1.63	m	2.23		31.39; 31.40 [2]	CH2
3'	1.36	m			29.08-29.83 [3]	CH2
4'	1.36	m			29.08-29.83 [3]	CH2
5'	1.36	m			29.08-29.83 [3]	CH2
6'	1.36	m			29.08-29.83 [3]	CH2
7'b	2.08	m			27.29; 27.27 [4]	CH2
8'b	5.41	m			128.05; 128.21; 130.03; 130.22 [6]	CH
9'b	5.41	m			128.05; 128.21; 130.03; 130.22 [6]	CH
10'b	2.83	t		5.8 (9'b ; 11'b)	25.71	CH2
11'b	5.41	m			128.05; 128.21; 130.03; 130.22 [6]	CH
12'b	5.41	m			128.05; 128.21; 130.03; 130.22 [6]	CH

13'b	2.08	m			27.29; 27.27 [4]	CH2
14'b	1.43	sx			22.88	CH2
15'b	0.96	t	0.68	7.5	13.92	CH3
**Cardanol with aliphatic chain C (triene; 36%)**						
1	-				155.33	C
2	6.70	s (b)	1.00		115.40	CH
3	-				145.00; 145.02 [1]	C
4	6.81	d	1.00	7.5	121.04	CH
5	7.18	t	1.00	7.7	129.47	CH
6	6.69	dd	1.00		112.58	CH
1'	2.59	t	2.00	7.7	35.89	CH2
2'	1.63	m	2.23		31.39; 31.40 [2]	CH2
3'	1.36	m			29.08-29.83 [3]	CH2
4'	1.36	m			29.08-29.83 [3]	CH2
5'	1.36	m			29.08-29.83 [3]	CH2
6'	1.36	m			29.08-29.83 [3]	CH2
7'c	2.08	m			27.29; 27.27 [4]	CH2
8'c	5.41	m			130.48	CH
9'c	5.41	m			127.63	CH
10'c	2.84	t		6.2 (11'c; 9'c)	25.63	CH2
11'c	5.47	m			126.86	CH
12'c	5.48	m			129.38	CH
13'c	2.88	t	0.77	6.1 (14'c; 12'c)	31.59	CH2
14'c	5.87	ddt	0.36	17.1 (15'E); 10.0 (15'Z); 6.1 (13')	136.91	CH
15'c	5.04 (Z)	dq	0.45	10.0 (14'); 1.7 (15'E); 1.7 (13')	114.82	CH2
15'c	5.10 (E)	dq	0.44	17.1 (14'); 1.7 (15'Z); 1.7 (13')	114.82	CH2

[1] ^{13}C signals of C3 of the compounds A, B, and C of cardanol: Assignment of the signals to the individual compounds is not possible.
[2] ^{13}C signals of C2' of the compounds A, B, and C of cardanol: Assignment of the signals to the individual compounds is not possible.
[3] ^{13}C signals of C3'-6' of the compounds A, B, an C and C11'a-12'a of cardanol: 29.08; 29.30; 29.31; 29.36; 29.38; 29.48; 29.49; 29.70; 29.73; 29.81; 29.83. Assignment of the signals to the individual positions and compounds is not possible.
[4] ^{13}C signals of C7' of the compounds B, and C, C10'a, and C13'b of cardanol: Assignment of the signals to the individual positions and compounds is not possible.
[5] ^{13}C signals of C8'a and C9'a of cardanol: Assignment of the signals to the individual positions is not possible.
[6] ^{13}C signals of C8'b, C9'B, C11'b and C12'b of cardanol. Assignment of the signals to the individual positions is not possible.

Supplementary Table S2: NMR correlations observed for component A (42%) in cardanol

Position Atom	δ 1H in ppm	H multiplicity	H integral	HH coupling in Hz	δ 13C in ppm	C multiplicity	HSQC correlation of H# with C#	HMBC correlation of H# with C#	HH COSY correlation with H#	HH-NOESY correlation with H#
1	/				155.33	C				
2	6.70	s (b)	1.00		115.40	CH	C2	C1'; C6; C4; C3*weak; C1	H1'; H4 *weak	H1'; H2'; H3'-6'
3	/				145.00; 145.02 [1]	C				
4	6.81	d	1.00	7.5	121.04	CH	C4	C1'; C6; C2; C5; C3*weak; C1*weak	H2; H5; H1'*weak	H5; H1'; H2'; H3'-6'
5	7.18	t	1.00	7.7	129.47	CH	C5	C6*weak; C2*weak; C4*weak; C3; C1	H6; H4	H6; H4
6	6.69	dd	1.00		112.58	CH	C6	C2; C4; C1	H5	H5
1'	2.59	t	2.00	7.7	35.89	CH2	C1'	C2; C4; C3; C2'; C3'-6'	H2'; H2; H4*weak	H3'-6'; H2'; H2; H4
2'	1.63	m	2.23		31.39; 31.40 [2]	CH2	C2'[2]	C1'; C3; C3'-6'	H3'-6'; H1'	H3'-6'; H1'; H2; H4
3'	1.36	m			29.08-29.83 [3]	CH2	C3'-6'a; C11'-C12'[3]	C7'; C3'-6'; C2'; C1'; C8'-9'	H2'; H7'	H1'; H2'; H7'; H8'-9'*weak; H2 *weak; H4*weak
4'	1.36	m			29.08-29.83 [3]	CH2	C3'-6'a; C11'-C12'[3]	C7'; C3'-6'; C2'; C1'; C8'-9'	H2'; H7'	H1'; H2'; H7'; H8'-9'*weak; H2 *weak; H4*weak
5'	1.36	m			29.08-29.83 [3]	CH2	C3'-6'; C11'-C12'[3]	C7'; C3'-6'; C2'; C1'; C8'-9'	H2'; H7'	H1'; H2'; H7'; H8'-9'*weak; H2 *weak; H4*weak
6'	1.36	m			29.08-29.83 [3]	CH2	C3'-6'; C11'-C12'[3]	C7'; C3'-6'; C2'; C1'; C8'-9'	H2'; H7'	H1'; H2'; H7'; H8'-9'*weak; H2 *weak; H4*weak
7'a	2.08	m			27.29; 27.27 [4]	CH2	C7'; C10'[4]	C3'-6'; C8'-9'	H3'-6'; H8'-9'	H3'-6'; H8'-9'
8'a	5.41	m			129.93; 130.05 [5]	CH	C8'-9'[5]	C7'/10'; C3'-6'/11'-12'	H7'/10'	H3'-6'/H11'-14'; H7‘/10'

9'a	5.41	m			129.93; 130.05 [5]	CH	C8'-9' [5]	C7'/10'; C3'-6'/11'-12'	H7'/10'	H3'-6'/H11'-14'; H7'/10'
10'a	2.08	m			27.29; 27.27 [4]	CH2	C7'; C10'[4]	C3'-6'/11'-12'; C8'-9'; C14'	H11'-14'; H8'-9'	H11'-14'; H8'-9'
11'a	1.36	m			29.08-29.83 [3]	CH2	C3'-6'; C11'-C12'[3]	C15'; C14'; C10'; C11'-12'; C13'; C8'-9'	H10'; H15'	H15'; H10'; H8'-9'*weak
12'a	1.36	m			29.08-29.83 [3]	CH2	C3'-6'; C11'-C12'[3]	C15'; C14'; C10'; C11'-12'; C13'; C8'-9'	H10'; H15'	H15'; H10'; H8'-9'*weak
13'a	1.36	m			31.87	CH2	C13'	C15'; C14'; C10'; C11'-12'; C13'; C8'-9'	H10'; H15'	H15'; H10'; H8'-9'*weak
14'a	1.36	m			22.76	CH2	C14'	C15'; C14'; C10'; C11'-12'; C13'; C8'-9'	H10'; H15'	H15'; 10'; H8'-9'*weak
15'a	0.94	t	1.28	7.1	14.23	CH3	C15'	C14'; C13'	H11'-14'	H11'-14'

[1] ^{13}C signals of C2 of the compounds A. B. and C in cardanol: Assignment of the signals to the individual compounds is not possible. [2] ^{13}C signals of C2' of the compounds A. B. and C of cardanol: Assignment of the signals to the individual compounds is not possible. [3] ^{13}C signals of C3'-6' of the compounds A. B. and C and C11'a-12'a of cardanol: 29.08; 29.30; 29.31; 29.36; 29.38; 29.48; 29.49; 29.70; 29.73; 29.81; 29.83 ppm. Assignment of the signals to the individual positions and compounds is not possible. [4] ^{13}C signals of C7' of the compounds A. B. and C. C10'a and C13'b: Assignment of the signals to the individual positions and compounds is not possible. [5] ^{13}C signals of C8'a and C9'a. Assignment of the signals to the individual positions is not possible.

Supplementary Table S3: NMR correlations observed for component B (22%) in cardanol

Position Atom	δ 1H in ppm	H multiplicity	H integral	HH coupling in Hz	δ 13C in ppm	C multiplicity	HSQC correltion of H# with C#	HMBC correlation of H# with C#	HH COSY correlation with H#	HH-NOESY correlation with H#
1	/				155.33	C				
2	6.70	s (b)	1.00		115.40	CH	C2	C1'; C6; C4; C3*weak; C1	H1'; H4 *weak	H1'; H2'; H3'-6'
3	/				145.00; 145.02 [1]	C				
4	6.81	d	1.00	7.5	121.04	CH	C4	C1'; C6; C2; C5; C3*weak; C1*weak	H2; H5; H1'*weak	H5; H1'; H2'; H3'-6'
5	7.18	t	1.00	7.7	129.47	CH	C5	C6*weak; C2*weak; C4*weak; C3; C1	H6; H4	H6; H4
6	6.69	dd	1.00		112.58	CH	C6	C2; C4; C1	H5	H5
1'	2.59	t	2.00	7.7	35.89	CH2	C1'	C2; C4; C3; C2'; C3'-6'	H2'; H2; H4*weak	H3'-6'; H2'; H2; H4
2'	1.63	m	2.23		31.39; 31.40 [2]	CH2	C2'[2]	C1'; C3; C3'-6'	H3'-6'; H1'	H3'-6'; H1'; H2; H4
3'	1.36	m			29.08-29.83 [3]	CH2	C3'-6'[3]	C7'; C3'-6'; C2'; C1'; C8'-9'	H2'; H7'	H1'; H2'; H7'; H8'-9'*weak; H2 *weak; H4*weak
4'	1.36	m			29.08-29.83 [3]	CH2	C3'-6'[3]	C7'; C3'-6'; C2'; C1'; C8'-9'	H2'; H7'	H1'; H2'; H7'; H8'-9'*weak; H2 *weak; H4*weak
5'	1.36	m			29.08-29.83 [3]	CH2	C3'-6'[3]	C7'; C3'-6'; C2'; C1'; C8'-9'	H2'; H7'	H1'; H2'; H7'; H8'-9'*weak; H2 *weak; H4*weak
6'	1.36	m			29.08-29.83 [3]	CH2	C3'-6'[3]	C7'; C3'-6'; C2'; C1'; C8'-9'	H2'; H7'	H1'; H2'; H7'; H8'-9'*weak; H2 *weak; H4*weak
7'b	2.08	m			27.29; 27.27 [4]	CH2	C7'; C13'b[4]	C3'-6'; C8'-9'	H3'-6'; H8'-9'	H3'-6'; H8'-9'; H10'

8'b	5.41	m			128.05; 128.21; 130.03; 130.22 [6]	CH	C8'-C9'/ C11'-C12'[6]	C10'; C7'; C3'-6'; C8'-9'/11'-12'	H7'; H10'	H3'-6'; H7'; H10'
9'b	5.41	m			128.05; 128.21; 130.03; 130.22 [6]	CH	C8'-C9'/ C11'-C12'[6]	C10'; C7'; C3'-6'; C8'-9'/11'-12'	H8'-9' (5.41): H7' (2.08); H10' (2.83)	H3'-6'; H7'; H10'
10'b	2.83	t		5.8 (9'b; 11'b)	25.71	CH2	C10'	C8'-9'/11'-12'[6]; C7'/13'	H8'-9'/11'-12'; H7'/H13'*very weak	H3'-6'; H7'/13'; H8'-9'/11'-12'
11'b	5.41	m			128.05; 128.21; 130.03; 130.22 [6]	CH	C8'-C9'/ C11'-C12'[6]	C 10'; C7'/13'; C8'-9'/11'-12'	H13'; H10'	H13'; H10'
12'b	5.41	m			128.05; 128.21; 130.03; 130.22 [6]	CH	C8'-C9'/ C11'-C12'[6]	C 10'; C7'/13'; C8'-9'/11'-12'	H13'; H10'	H13'; H10'
13'b	2.08	m			27.29; 27.27 [4]	CH2	C7'; C13'b[4]	C15'; C14'; C8'-9'/11'-12'	H14'; H11'-12'; H10'*very weak	11'-12'; H10'
14'b	1.43	sx			22.88	CH2	C14'	C15'	H13'; H15'	H15'
15'b	0.96	t	0.68	7.5	13.92	CH3	C15'	C14'	H14'	H14'

[6] ^{13}C signals of C8'b. C9'B. C11'b and C12'b: Assignment of the signals to the individual positions is not possible.

Supplementary Table S4: NMR correlations observed for component C (36%) in cardanol

Position Atom	δ 1H in ppm	H multiplicity	H integral	HH coupling in Hz	δ 13C in ppm	C multiplicity	HSQC correltion of H# with C#	HMBC correlation of H# with C#	HH COSY correlation with H#	HH-NOESY correlation with H#
1	/				155.33	C				
2	6.70	s (b)	1.00		115.40	CH	C2	C1'; C6; C4; C3*weak; C1	H1'; H4 *weak	H1'; H2'; H3'-6'
3	/				145.00; 145.02 [1]	C				
4	6.81	d	1.00	7.5	121.04	CH	C4	C1'; C6; C2; C5; C3*weak; C1*weak	H2; H5; H1'*weak	H5; H1'; H2'; H3'-6'
5	7.18	t	1.00	7.7	129.47	CH	C5	C6*weak; C2*weak; C4*weak; C3; C1	H6; H4	H6; H4
6	6.69	dd	1.00		112.58	CH	C6	C2; C4; C1	H5	H5
1'	2.59	t	2.00	7.7	35.89	CH2	C1'	C2; C4; C3; C2'; C3'-6'	H2'; H2; H4*weak	H3'-6'; H2'; H2; H4
2'	1.63	m	2.23		31.39; 31.40 [2]	CH2	C2'[2]	C1'; C3; C3'-6'	H3'-6'; H1'	H3'-6'; H1'; H2; H4
3'	1.36	m			29.08-29.83 [3]	CH2	C3'-6'	C7'; C3'-6'; C2'; C1'; C8'	H2'; H7'	H1'; H2'; H7'; H8'-9'*weak; H2*weak; H4*weak
4'	1.36	m			29.08-29.83 [3]	CH2	C3'-6'	C7'; C3'-6'; C2'; C1'; C8'	H2'; H7'	H1'; H2'; H7'; H8'-9'*weak; H2*weak; H4*weak
5'	1.36	m			29.08-29.83 [3]	CH2	C3'-6'	C7'; C3'-6'; C2'; C1'; C8'	H2'; H7'	H1'; H2'; H7'; H8'-9'*weak; H2*weak; H4*weak
6'	1.36	m			29.08-29.83 [3]	CH2	C3'-6'	C7'; C3'-6'; C2'; C1'; C8'	H2'; H7'	H1'; H2'; H7'; H8'-9'*weak; H2*weak; H4*weak

7'c	2.08	m			27.29; 27.27 [4]	CH2	C3'-6'	C3'-6'; C9'; C8'	H3'-6'; H8'-9'; H10'*very weak	H3'-6'; H8'-9'; H10'
8'c	5.41	m			130.48	CH	C8'	C10'; C7'; C3'-6'; C12*weak	H7'; H10'	H3'-6'; H7'; H10'
9'c	5.41	m			127.63	CH	C9'	C10'; C7'; C3'-6'; C12*weak	H7'; H10'	H3'-6'; H7'; H10'
10'c	2.84	t		6.2 (11'c; 9'c)	25.63	CH2	C10'	C11'; C9'; C12'; C8'; 7'C*weak	H8'-9'; H11'; H12'; H7'*very weak	H3'-6'; H7'; H8'-9'; H11'; H12'
11'c	5.47	m			126.86	CH	C11'	C10'; C13'; C9'*weak	H10'; H13'	H10'
12'c	5.48	m			129.38	CH	C12'	C13'; C14'; C10'	H10'; H13'	H13'
13'c	2.88	t	0.77	6.1 (14'c; 12'c)	31.59	CH2	C13'	C15'; C11'; C12'; C14'; C9'*weak	H14'; H15'Z; H15'E; H11'; H12'	H15'Z; H15'E; H12'; H14'
14'c	5.87	ddt	0.36	17.1 (15'E); 10.0 (15'Z); 6.1 (13')	136.91	CH	C14'	C13'; C15'*weak; C11'	H13'; H15'Z; H15'E	H13'; H15'Z; H15'E
15'c	5.04 (Z)	dq	0.45	10.0 (14'); 1.7 (15'E); 1.7 (13')	114.82	CH2	C15'	C13'; C14'*weak	H14'; H13'	H13'; H14'
15'c	5.10 (E)	dq	0.44	17.1 (14'); 1.7 (15'Z); 1.7 (13')	114.82	CH2	C15'	C13'; C14'	H14'; H13'	H13'; H14'
